# Potentially pathogenic free-living amoebae at very high altitude: Detection by multiplex qPCR in the Northern Altiplano fascioliasis hyperendemic area in Bolivia

**DOI:** 10.1016/j.onehlt.2025.100985

**Published:** 2025-02-08

**Authors:** Patricia Pérez-Pérez, Patricio Artigas, María Reyes-Batlle, Elizabeth Córdoba-Lanús, Rubén L. Rodríguez-Expósito, Pablo F. Cuervo, Angélica Domínguez-de-Barros, Omar García-Pérez, M. Adela Valero, Alejandra De Elías, René Anglés, Santiago Mas-Coma, José E. Piñero, M. Dolores Bargues, Jacob Lorenzo-Morales

**Affiliations:** a Instituto Universitario de Enfermedades Tropicales y Salud Pública de Canarias (IUETSPC), Universidad de La Laguna (ULL), San Cristóbal de La Laguna, Spain; b Departamento de Obstetricia y Ginecología, Pediatría, Medicina Preventiva y Salud Pública, Toxicología, Medicina Legal y Forense y Parasitología, Universidad de La Laguna, San Cristóbal de La Laguna, Spain; c CIBER de Enfermedades Infecciosas, Instituto de Salud Carlos III, C/ Monforte de Lemos 3-5. Pabellón 11. Planta 0, 28029 Madrid, Spain; d Departamento de Parasitología, Facultad de Farmacia, Universidad de Valencia, Av. Vicent Andrés Estellés s/n, 46100 Burjassot, Valencia, Spain; e Cátedra de Parasitología, Facultad de Medicina, Universidad Mayor de San Andrés (UMSA), Av. Saavedra, Miraflores, La Paz, Bolivia

**Keywords:** Free-living amoebae, *Vermamoeba vermiformis*, *Acanthamoeba*, *Balamuthia mandrillaris*, *Naegleria fowleri*, Multiplex q-PCR, Very high altitude, Hyperendemic area of human fascioliasis, Bolivia

## Abstract

Free-living amoebae (FLA), which are frequently found in the environment, include opportunistic pathogenic genera/species such as *Acanthamoeba* spp., *Balamuthia mandrillaris*, *Naegleria fowleri* and *Vermamoeba vermiformis*. These pathogenic FLA are causative agents of amoebic encephalitis and keratitis in the case of *Acanthamoeba* genus and *V. vermiformis*. In addition, amoebic infections are often related to contamination of domestic and recreational water sources. This study aimed to identify potentially pathogenic FLA in the hyperendemic area of human fascioliasis in a very-high-altitude area (3800–4100 m a.s.l.) of Bolivia and examine whether an association between both pathogens could be established from the environmental point of view. A total of 55 samples (28 soil and 27 water samples) were collected from various locations in the Northern Altiplano of Bolivia. Samples were processed by multiplex qPCR to detect the four pathogenic FLA genera/species. All samples were positive for the presence of *V. vermiformis*, followed by *Acanthamoeba* spp. which was positive in 18 soil and 10 water samples. In contrast, *B. mandrillaris* was only detected in soil sources, whereas *N. fowleri* was not detected in any of the samples. The coexistence and diverse distribution of multiple FLA species in many locations at such a high altitude is worth mentioning and indicates a potential risk of coinfections. These findings suggest that FLA surveillance is a crucial factor to be considered when implementing preventive measures and improving public health in fascioliasis hyperendemic areas.

## Highlights

• The presence of FLA in very high-altitude areas such as the Northern Bolivian Altiplano is demonstrated.
• *Vermamoeba vermiformis* and *Acanthamoeba* genus were the most frequent FLA in the samples included in this study.
• FLA not only adapt to extreme environmental conditions but also expand in a way similar as fascioliasis vectors.
• Evidencing a parallelism in the dispersion of both pathogens which should raise awareness in the health systems.
• Overall, these unique environmental conditions and the detection of FLA should be urgently considered in the region.

## Graphical abstract

**Figure:**
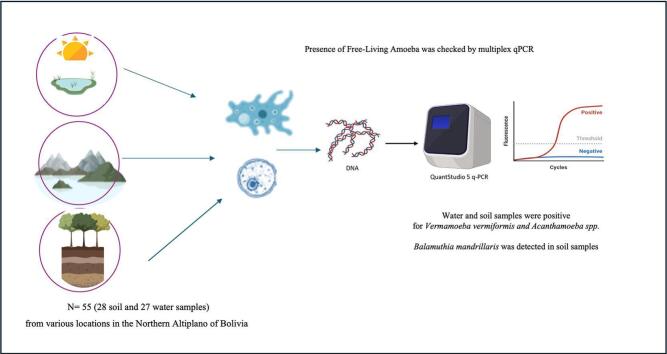

## 1 Introduction

Protozoa belonging to the genus *Acanthamoeba* and the species *Naegleria fowleri*, *Balamuthia mandrillaris*, *Sappinia pedata* and *Vermamoeba vermiformis* are opportunistic pathogens [[1], [2], [3]]. These parasites are classified as free-living amoebae (FLA) and can affect the Central Nervous System (CNS), causing encephalitis, as well as disseminated infections, primarily involving the skin [2]. In addition, *Acanthamoeba* and *Vermamoeba vermiformis* have been reported as causative agents of amoebic keratitis [3].

These opportunistic pathogenic protozoa are widely distributed in the environment. They have been detected in sources, including soil, fresh and brackish water, wastewater, hot springs or dust [2]. In this sense, FLA have been isolated in diverse settings such as vegetables, cooling towers, air conditioning systems, dental water supplies and contact lenses [1,4,5]. A significant public health concern related to FLA is their ability to act as reservoirs for other pathogenic microorganisms [4,6]. Therefore, it is crucial to study the ecological niches of FLA and their capacity to colonize areas associated with human activity [7].

Previous studies have routinely assessed the prevalence and diversity of FLA in environmental and clinical samples using culture methods and conventional PCR. However, a multiplex q-PCR for the four previously mentioned pathogenic FLA has been introduced as a highly efficient and rapid molecular technique. This method is particularly effective for detecting FLA in different sources due to its exceptional sensitivity and specificity [8,9].

The Northern Bolivian Altiplano (NBA) is recognized as a hyperendemic region for fascioliasis, with the highest reported prevalence and infection intensities in the human population [[10], [11], [12], [13]]. Within this region, children and women are the most affected demographic groups, which poses significant challenges to community development [14]. Furthermore, this situation highlights social issues, notably those related to gender dynamics within the Aymara communities that-inhabit the region [15].

The severe impact of the hyperendemic situation in the NBA arises from several factors. First, the disease's high pathogenicity and associated morbidity in humans [16,17] contribute significantly to its detrimental effects. Second, fascioliasis induces immune suppression [[18], [19], [20], [21], [22]], which increases vulnerability to coinfections withother pathogenic parasites [10,11], as well as bacteria and viruses [23,24]. Third, the disease has substantial veterinary implications, as it affects livestock critical to human subsistence in rural areas at such very high altitudes [25]. Given these factors, fascioliasis stands out as the disease with the most severe adverse impact on public health in the NBA.

In response to this public health challenge, a comprehensive One Health initiative has been implemented to reduce infection and reinfection rates. This multidisciplinary approach integrates annual preventive chemotherapy campaigns with continuous monitoring to evaluate its effectiveness over time [[26], [27], [28], [29], [30], [31], [32], [33], [34]].

The numerous sources of human infection of fascioliasis include the ingestion of metacercaria from various sources, such as freshwater and semi-aquatic plants, as well as terrestrial plants that require frequent irrigation. Additionally, traditional local dishes made from sylvatic plants, and vegetables sold in uncontrolled urban markets are common sources of infection. Other routes of transmission include the consumption of contaminated natural water, beverages, juices, soups, and dishes made with untreated water, as well as the washing of kitchen utensils, vegetables, fruits, and tubers with contaminated water [35]. The connection between these sources of infectionand the cultural and social practices of the local populations of each region [15], in addition the effects of climate and global warming could be relevant to this concern [[36], [37], [38]].

To the knowledge of the authors, there is no evidence establishing a direct relationship between this type of trematodiasis and FLA. However, both infections may occur in the same geographical area, especially in regions with poor sanitary conditions. Hence, this research aims to determine, for the first time, the presence of FLA in water and soil samples from a hyperendemic region of human fascioliasis using a multiplex qPCR assay. Likewise, we seek to analyze whether the distribution pattern of these FLA is linked to the factors that influence the eco-epidemiological distribution of this food-borne disease in the NBA. Besides this, the high altitude of the endemic area provides an opportunity to assess the ability of these FLA to adapt to the extreme environmental and climatic conditions typical of such altitude (the threshold between high altitude and very high altitude is generally considered to be 3200 m a.s.l. - above sea level).

## 2 Materials and methods

### 2.1 Area of study

**Fig. 1:**
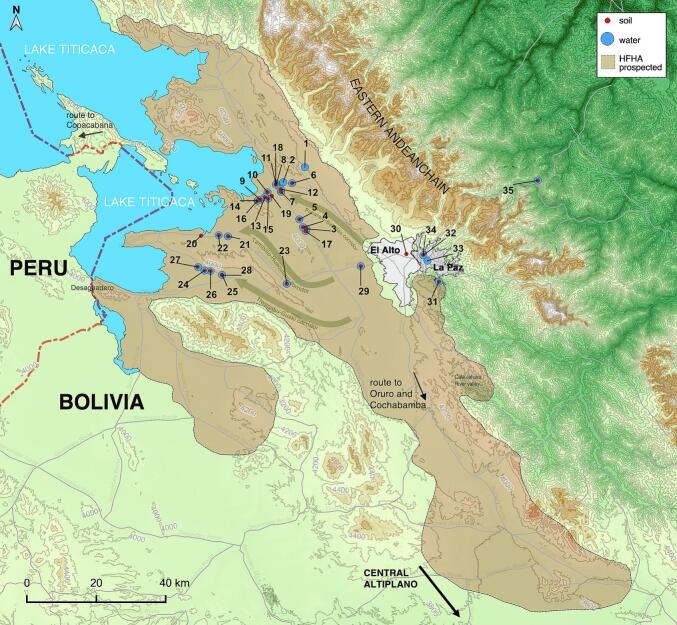
Map of the Northern Bolivian Altiplano human fascioliasis hyperendemic area (HFHA), located between Lake Titicaca and the city of El Alto and the valley of La Paz city, showing the distribution of the sampling locations for free-living amoebae. Coordinates and altitudes of each site are indicated in Table 1. Red circles = soil sampling locations; blue circles = water sampling locations; light brown shaded area = HFHA prospected zone [[13], [15], [29]]. (For interpretation of the references to colour in this figure legend, the reader is referred to the web version of this article.)

Field surveys were conducted across the extensive region of the NBA, situated between 3820 and 4100 m a.s.l., eastward from Lake Titicaca and up to the cities of El Alto and La Paz. The sampling tasks were carried out inside the boundaries of the fascioliasis human hyperendemic area [13,15,29] including the three main flatland corridors, separated by hilly chains (Fig. 1). The surveys of both water and soil were carried out in localities with previously analyzed freshwater collections which were positive for the presence of lymnaeid snail vectors of fascioliasis. In addition, several human communities, villages or cities in which human fascioliasis infection cases were detected, were also surveyed (Fig. 1).

### 2.2 DNA extraction from soil and water samples

Fifty-five environmental samples were collected: 28 from soil and 27 from water sources through the Northern Bolivian Altiplano (Table 1, Fig. 1). 15 mL sterile tubes were used to take soil and water samples, which were kept at 4 °C until their processing. Each soil sample was dissolved in 10 mL of Page's Amoeba solution (PAS) after being weighed at 0.6 g. The soil suspension was highly vortexed for 5 min and approximately 1 mL of the supernatant was added to the Maxwell^Ⓡ^ RSC (Promega, Madrid, Spain) [39], following the manufacturer's instructions. On the other hand, each water sample was centrifuged and 1 mL of the pellet was placed directly in the Maxwell^Ⓡ^ RSC too. After the procedure, approximately 200 μL of purified DNA was obtained and conserved for further analysis by q-PCR.

**Table 1:** Coordinates, altitudes and type of habitats of the localities in the Northern Bolivian Altiplano from where fresh water sources and environmental soil samples were analyzed for FLA detection.

**No. In Map**⁎	**Localities (description)**	**Water**	**Soil**	**Corridor/area**	**Municipality**	**Province**	**Habitat**	**Coordinates**	**Altitude (m)**
1	Suriquiña (farmhouse)	+	−	Pucarani-Batallas	Batallas	Los Andes	rural	16°15′24.48”S/68°27′30.54”W	4001
2	Karawisa river (meander)	+	−	Pucarani-Batallas	Batallas	Los Andes	rural	16°17′51.43”S/68°31′03.11”W	3870
3	Ancocagua (St. Gloria bridge)	+	+	Pucarani-Batallas	Pucarani	Los Andes	rural	16°25′26.83”S/68°27′18.25”W	3853
4	Pucarani (wetland)	+	+	Pucarani-Batallas	Pucarani	Los Andes	rural	16°25′11.08”S/68°27′49.15”W	3850
5	Pucarani (irrigation canal)	+	+	Pucarani-Batallas	Pucarani	Los Andes	rural	16°23′54.02”S/68°28′24.60”W	3859
6	Chirapaca (school yard)	+	+	Pucarani-Batallas	Batallas	Los Andes	rural	16°18′00.89”S/68°29′34.99”W	3899
7	Cullucachi/ Calasaya (irrigation canal)	+	+	Pucarani-Batallas	Batallas	Los Andes	rural	16°19′24.50”S/68°31′24.26”W	3860
8	Batallas (rural area near homes)	+	+	Pucarani-Batallas	Batallas	Los Andes	rural	16°18′07.39”S/68°32′09.27”W	3850
9	Cutusuma/ Tacanoque (wetland)	+	+	Pucarani-Batallas	Puerto Pérez	Los Andes	rural	16°20′42.55”S/68°34′43.28”W	3830
10	Cutusuma (wetland)	+	+	Pucarani-Batallas	Puerto Pérez	Los Andes	rural	16°19′29.17”S/68°33′36.74”W	3840
11	Batallas (irrigation canal)	+	−	Pucarani-Batallas	Batallas	Los Andes	rural	16°18′14.37”S/68°32′13.91”W	3849
12	Cullucachi (school yard)	+	+	Pucarani-Batallas	Puerto Pérez	Los Andes	rural	16°18′58.50”S/68°31′28.31”W	3845
13	Cutusuma (school yard)	+	+	Pucarani-Batallas	Puerto Pérez	Los Andes	rural	16°20′17.05”S/68°34′00.84”W	3837
14	Khanapata (health centre)	−	+	Pucarani-Batallas	Puerto Pérez	Los Andes	rural	16°20′51.14”S/68°35′13.52”W	3842
15	Chijipata Baja (school yard)	+	+	Pucarani-Batallas	Batallas	Los Andes	rural	16°20′30.83”S/68°33′28.98”W	3841
16	Khanapata (school yard)	+	−	Pucarani-Batallas	Puerto Pérez	Los Andes	rural	16°20′48.26”S/68°35′08.58”W	3842
17	Ancocagua (school yard)	+	+	Pucarani-Batallas	Pucarani	Los Andes	rural	16°25′52.17”S/68°27′20.09”W	3866
18	Batallas (pigsty)	−	+	Pucarani-Batallas	Batallas	Los Andes	rural	16°18′13.87”S/68°32′15.39”W	3849
19	Chijipata Alta (school yard)	−	+	Pucarani-Batallas	Batallas	Los Andes	rural	16°20′04.26”S/68°32′54.10”W	3841
20	Huacullani (school yard)	−	+	Tambillo-Huacullani	Tiawanaku	Ingavi	rural	16°26′33.90”S/68°44′26.30”W	3830
21	Lacaya (wetland)	+	+	Tambillo-Huacullani	Pucarani	Los Andes	rural	16°26′39.29”S/68°40′02.73”W	3822
22	Chojasivi (drinking trough)	+	+	Tambillo-Huacullani	Pucarani	Los Andes	rural	16°26′28.17”S/68°41′33.65”W	3848
23	Tambillo (drainage ditch)	+	+	Tambillo-Huacullani	Laja	Los Andes	rural	16°34′23.74”S/68°30′28.43”W	3871
24	Yanarico (school yard)	+	+	Tiwanaku-Guaqui	Tiwanaku	Ingavi	rural	16°31′37.43”S/68°45′01.05”W	3847
25	Tiwanaku (drinking trough)	+	+	Tiwanaku-Guaqui	Tiwanaku	Ingavi	rural	16°32′56.21”S/68°41′00.70”W	3855
26	Achuta Grande (school yard)	+	+	Tiwanaku-Guaqui	Tiwanaku	Ingavi	rural	16°32′16.84”S/68°42′52.93”W	3863
27	Chambi Grande (school yard)	−	+	Tiwanaku-Guaqui	Tiwanaku	Ingavi	rural	16°32′13.91”S/68°43′51.37”W	3858
28	Tiwanaku (health centre)	−	+	Tiwanaku-Guaqui	Tiwanaku	Ingavi	suburban	16°32′59.74”S/68°40′59.89”W	3857
29	Veterinary College (drainage canal)	+	+	El Alto	Laja	Los Andes	suburban	16°31′32.08”S/68°18′24.40”W	3905
30	El Alto, La Paz station (square)	−	+	El Alto	El Alto	Murillo	urban	16°29′32.96”S/68°10′58.27”W	4067
31	La Paz, Valle de la luna (park)	+	−	La Paz	La Paz	Murillo	suburban	16°34′01.81”S/68°05′40.43”W	3297
32	La Paz, Plaza San Francisco (square)	+	−	La Paz	La Paz	Murillo	urban	16°29′45.04”S/68°08′12.82”W	3622
33	La Paz, Plaza Abaroa (square)	+	−	La Paz	La Paz	Murillo	urban	16°30′37.34”S/68°07′32.52”W	3539
34	La Paz, Mercado Lanza (square)	−	+	La Paz	La Paz	Murillo	urban	16°29′40.31”S/68°08′14.04”W	3630
35	Velo de la novia (Natural Park)	+	+	NP Cotapata	Coroico	Nor Yungas	natural park	16°17′34.22”S/67°49′33.99”W	3118

⁎ For numbers of localities see map in Fig. 1.

### 2.3 Multiplex real-time PCR assay (q-PCR)

Primers and the adapted TaqMan probes used in the multiplex q-PCR assay were adapted from the previously described assay by Qvarnstrom et al. [40], and Córdoba Lanús et al., [9], are listed in Table 2.

**Table 2:** Primers used in the qPCR for the detection of the free-living amoebae (FLA) in this study.

**FLA species**	**Primers sequence**	**DNA fragment (bp)**
*Acanthamoeba* spp.⁎	AcantF900 (5`-CCC AGA TCG TTT ACC GTG AA-3`) AcantR1100 (5`-TAA ATA TTA ATG CCC CCA ACT ATC C-3`) AcantProb (5`-JUN-CT GCC ACC GAA TAC ATT AGC ATG G-QSY-3`)	180
*Naegleria fowleri*⁎	NaeglF192 (3`-GTG CTG AAA CCT AGC TAT TGT AAC TCA GT-5`) NaeglR344 (5`-CAC TAG AAA AAG CAA ACC TGA AAG G-3`) NeglProb (5`-VIC-AT AGC AAT ATA TTC AGG GGA GCT GGG C-QSY-3`)	153
*Balamuthia mandrillaris*⁎	BalaF1451 (5`-TAA CCT GCT AAA TAG TCA TGC CAA T-3`) BalaR1621 (5`-CAA ACT TCC CTC GGC TAA TCA-3`) BalaProb (5`-6FAM-AG TAC TTC TAC CAA TCC AAC CGC CA-QSY-3`)	171
*Vermamoeba vermiformis*	Hv1227F (5′-TTA CGA GGT CAG GAC ACT GT- 3′)⁎⁎ VermRv (5´ TGCCTCAAACTTCCATTCGC 3′)⁎⁎⁎ VermProb (5`-ABI-TTG ATT CAG TGG GTG GTG GT-QSY-3`)⁎⁎⁎	235

⁎ Primers sequence described in [40]

⁎⁎ Forward primer sequence adapted from a previous study and described in [9].

⁎⁎⁎ This reverse primer sequence was specially designed in [9].

The q-PCR reactions were performed in a 10 μL final volume, using 10× TaqMan® Multiplex Master Mix (Applied Biosystems, ThermoFisher Scientific), 0.5 μM of each primer, 0.25 μM of the probe and 2 μL of the obtained DNA of each environmental sample. The q-PCR reaction was set up in a QuantStudio 5 real-time PCR machine (ThermoFisher Scientific, MA, USA) under the following conditions: a denaturalization step of 3 min at 95 °C; followed by 40 cycles consisting of two steps of 95 °C 15 s and 60 °C 1 min. The cycle threshold (Ct) was obtained by the software Design & Analysis v2.4.3 (ThermoFisher Scientific), and the detection limit of the amplicons for the 4 FLA ranged from Ct of 34 to 35, based on the previous results obtained by comparison with the DNA standard curves [9]. DNA from axenic cultures of *Acanthamoeba castellanii* Neff (ATCC®30011™), *Naegleria fowleri* (ATCC®30808™), an environmental isolate H3 of *Balamuthia mandrillaris* [41] and *Vermamoeba vermiformis* (NCBI MT320010) [42] were used as positive controls.

## 3 Results

The q-PCR assays detected the presence of pathogenic FLA in all the evaluated samples (Table 3, Table 4). The most prevalent FLA was *Vermamoeba vermiformis*, which was observed in 100 % of the samples (27 water +28 soil samples). From the total of the 55 samples, *Acanthamoeba* spp. was identified in 28 (50.9 %) samples of which, 18/28 (64.3 %) corresponded to soil and 10/27 (37 %) to water samples. Moreover, *Balamuthia mandrillaris* was only identified in soil sources (10/28; 35.7 %) while *Naegleria fowleri* was not detected in any of the analyzed samples.

**Table 3:** FLA species identified by qPCR in fresh water sources from the Northern Bolivian Altiplano. *Ac* = *Acanthamoeba* spp.; *Nf = Naegleria fowleri; Bm = Balamuthia mandrillaris; Vv = Vermamoeba vermiformis.* The number of the water sample corresponds to its geographical location (see Table 1 and Fig. 1, Fig. 2). *Ac, Nf, Bm & Vv values (Values are given as the cycle threshold (Ct).*

**Water** **sample**	**Locality (description)**	**Corridor/area**	**qPCR**			
			***Ac***	***Nf***	***Bm***	***Vv***
1	Suriquiña (farmhouse)	Pucarani-Batallas	–	–	–	31.19
2	Karawisa river (meander)	Pucarani-Batallas	–	–	–	31.48
3	Ancocagua (St. Gloria bridge)	Pucarani-Batallas	35.00	–	–	31.20
4	Pucarani (wetland)	Pucarani-Batallas	34.90	–	–	31.87
5	Pucarani (irrigation canal)	Pucarani-Batallas	–	–	–	31.38
6	Chirapaca (school yard)	Pucarani-Batallas	–	–	–	31.47
7	Cullucachi/ Calasaya (irrigation canal)	Pucarani-Batallas	–	–	–	31.92
8	Batallas (rural area near homes)	Pucarani-Batallas	–	–	–	31.17
9	Cutusuma/ Tacanoque (wetland)	Pucarani-Batallas	32.10	–	–	31.56
10	Cutusuma (wetland)	Pucarani-Batallas	–	–	–	31.84
11	Batallas (irrigation canal)	Pucarani-Batallas	34.50	–	–	31.71
12	Cullucachi (school yard)	Pucarani-Batallas	–	–	–	31.67
13	Cutusuma (school yard)	Pucarani-Batallas	31.54	–	–	31.53
15	Chijipata Baja (school yard)	Pucarani-Batallas	30.36	–	–	32.04
16	Khanapata (school yard)	Pucarani-Batallas	34.21	–	–	31.87
17	Ancocagua (school yard)	Pucarani-Batallas	–	–	–	30.50
21	Lacaya (wetland)	Tambillo-Huacullani	34.55	–	–	31.44
22	Chojasivi (drinking trough)	Tambillo-Huacullani	–	–	–	31.34
23	Tambillo (drainage ditch)	Tambillo-Huacullani	34.94	–	–	31.32
24	Yanarico (school yard)	Tiawanaku-Guaqui	–	–	–	31.65
25	Tiwanaku (drinking trough)	Tiawanaku-Guaqui	–	–	–	31.78
26	Achuta Grande (school yard)	Tiawanaku-Guaqui	–	–	–	31.25
29	Veterinary College (drainage canal)	El Alto	–	–	–	31.48
31	La Paz, Valle de la luna (park)	La Paz	–	–	–	31.50
32	La Paz, Plaza San Francisco (city)	La Paz	34.77	–	–	31.33
33	La Paz, Plaza Abaroa (city square)	La Paz	–	–	–	31.26
35	Velo de la novia (Natural Park)	NP Cotapata	–	–	–	31.56

**Table 4:** FLA species identified by qPCR in soil sources from the Northern Bolivian Altiplano. *Ac* = *Acanthamoeba* spp.; *Nf = Naegleria fowleri; Bm = Balamuthia mandrillaris; Vv = Vermamoeba vermiformis.* The number of the soil sample corresponds to its geographical location (see Table 1 and Fig. 1, Fig. 2). Shadowed areas show localities where the simultaneous presence of three FLA species was confirmed. *Ac, Nf, Bm & Vv values (Values are given as the cycle threshold (Ct)).*

**Soil** **sample**	**Localities (description)**	**Corridor/area**	**qPCR**			
			***Ac***	***Nf***	***Bm***	***Vv***
3	Ancocagua (St. Gloria bridge)	Pucarani-Batallas	–	–	–	33.98
4	Pucarani (wetland)	Pucarani-Batallas	–	–	–	30.74
5	Pucarani (irrigation canal)	Pucarani-Batallas	33.20	–	–	32.36
6	Chirapaca (school yard)	Pucarani-Batallas	32.80	–	32.20	29.40
7	Cullucachi/ Calasaya (irrigation canal)	Pucarani-Batallas	33.50	–	33.15	29.66
8	Batallas (rural area near homes)	Pucarani-Batallas	32.10	–	31.40	28.98
9	Cutusuma/ Tacanoque (wetland)	Pucarani-Batallas	–	–	–	30.17
10	Cutusuma (wetland)	Pucarani-Batallas	32.40	–	–	30.65
12	Cullucachi (school yard)	Pucarani-Batallas	33.75	–	–	29.80
13	Cutusuma (school yard)	Pucarani-Batallas	–	–	–	32.61
14	Khanapata (health centre)	Pucarani-Batallas	33.16	–	30.29	32.59
15	Chijipata Baja (school yard)	Pucarani-Batallas	–	–	30.73	30.00
17	Ancocagua (school yard)	Pucarani-Batallas	–	–	–	32.51
18	Batallas (pigsty)	Pucarani-Batallas	–	–	–	27.41
19	Chijipata Alta (school yard)	Pucarani-Batallas	33.40	–	30.75	32.71
20	Huacullani (school yard)	Tambillo-Huacullani	33.33	–	–	28.15
21	Lacaya (wetland)	Tambillo-Huacullani	33.10	–	–	29.59
22	Chojasivi (drinking trough)	Tambillo-Huacullani	34.20	–	–	33.75
23	Tambillo (drainage ditch)	Tambillo-Huacullani	34.00	–	34.30	29.90
24	Yanarico (school yard)	Tiawanaku-Guaqui	–	–	31.96	31.90
25	Tiwanaku (drinking trough)	Tiawanaku-Guaqui	–	–	–	34.13
26	Achuta Grande (school yard)	Tiawanaku-Guaqui	33.10	–	–	32.60
27	Chambi Grande (school yard)	Tiawanaku-Guaqui	32.35	–	–	31.54
28	Tiwanaku (health centre)	Tiawanaku-Guaqui	34.05	–	33.18	28.95
29	Veterinary College (drainage canal)	El Alto	34.65	–	–	31.36
30	El Alto, La Paz station (city square)	El Alto	–	–	–	33.51
34	La Paz, Mercado Lanza (city square)	La Paz	34.05	–	30.17	32.96
35	Velo de la novia (Natural Park)	NP Cotapata	32.50	–	–	30.59

The Ct values obtained by the qPCR reaction oscillated between 30.4 and 35 in water samples and 27.4 to 34.6 in soil samples for all detected FLA. Considering the previous results reported by Córdoba-Lanús et al., (2024), the DNA detected in only 0.6 g of the analyzed soil samples would correspond to 100^−1^ and 10^−1^ amoebae respectively.

**Fig. 2:**
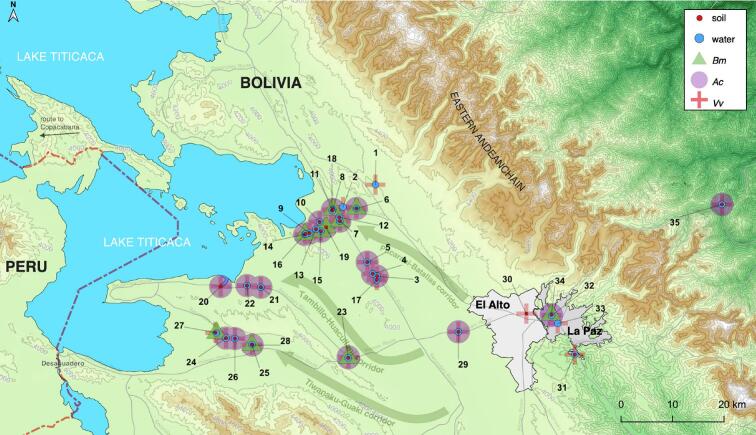
Map of the Northern Bolivian Altiplano human fascioliasis hyperendemic area showing the locations where free-living amoeba were detected. Coordinates and altitudes of each site are indicated in Table 1. Green triangles = *Balamuthia mandrillaris*; purple circle = *Acanthamoeba* spp.; red crosses = *Vermamoeba vermiformis.* Red circles = soil sampling locations; blue circles = water sampling locations. (For interpretation of the references to colour in this figure legend, the reader is referred to the web version of this article.)

**Fig. 3:**
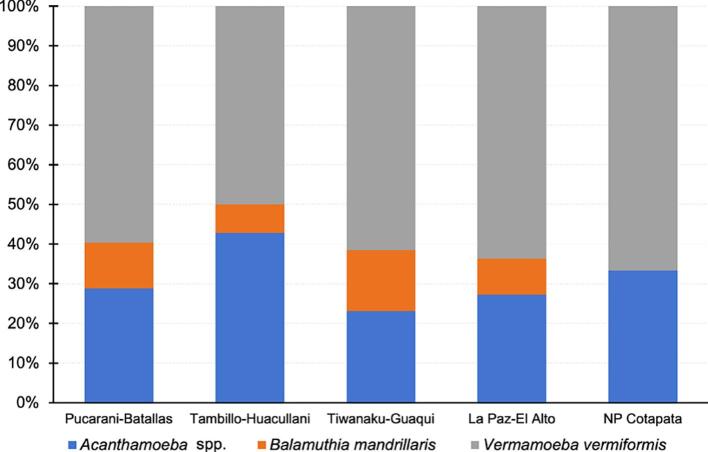
Geographical distribution of detected FLA according to the three main corridors of the fascioliasis hyperendemic zone.

Regarding geographical distribution, according to the three main corridors of the fascioliasis hyperendemic zone (Fig. 2), it has been observed that (Fig. 3):

a) In the Pucarani-Batallas corridor, the 3 species of free-living amoebae (*Acathamoeba* spp. *B. mandrillaris* and *V. vermiformis)* were identified in the localities analyzed, including especially rural or semi-urban habitats such as schools and health centers. In the 31 samples from this corridor (16 water; 15 soil), *Acanthamoeba* spp. was present in 15 (48.4 %), of which 7 (46.7 %) were water and 8 (53.3 %) were soil samples; *B. mandrillaris* was present only in 6 (53.3 %) soil samples*;* and *V. vermiformis* was identified in all samples, both water and soil (31/31, 100 %). It is worth mentioning that the simultaneous co-existence of 3 species of FLA, has been identified in 5 soil samples (5/31, 16.1 %) from this corridor, corresponding to the localities of Chirapaca (school yard), Cullucachi/ Calasaya (irrigation canal), Batallas rural village, Khanapata (health centre) and Chijipata Alta (school yard). The combination of two species of FLA was confirmed in 11 samples (11/31, 35.5 %) and the presence of only one species of FLA was observed in 15 samples (15/31, 48.4 %) (Table 3, Table 4 and Fig. 2).
b) In the Tambillo-Huacullani corridor, the 7 samples studied (3 water and 4 soil) allowed us to identify the 3 species of free-living amoebae (*Acanthamoeba* spp., *B. mandrillaris* and *V. vermiformis). Acanthamoeba* spp. was present in 6 of them (85.7 %): 2 (33.3 %) were water and 4 (66.6 %) were soil samples. *B. mandrillaris* was present only in 1 soil sample (14.3 %)*;* and *V. vermiformis* was identified in all samples, both water and soil (7/7, 100 %) (Table 3, Table 4 and Fig. 2). The simultaneous presence of the 3 species of FLA, was confirmed in only one locality, the rural village of Tambillo, in a drainage ditch, whereas the combination of two or only one species of FLA was confirmed in 5 samples (2 water, 3 soil; 5/7, 71.4 %), and in only 1 water sample (1/7, 14.3 %), respectively (Table 3, Table 4 and Fig. 2).
c) In the Tiwanaku-Guaqui corridor, similar results were obtained to those of the first and second corridors, confirming the presence of the same three FLA species. In the 8 samples analyzed (3 water and 5 soil), *Acanthamoeba* spp. was present in 3 soil samples (37.5 %), *B. mandrillaris* in 2 soil samples (25 %)*,* and *V. vermiformis* in all samples, both water and soil (8/8, 100 %). The simultaneous presence of 3 species of FLA, has been identified in only one soil sample (1/8, 12.5 %) and corresponding to the locality of Tiwanaku (health centre). The combination of two species of FLA was confirmed in 3 soil samples (3/8, 37.5 %) and corresponding to the rural localities of Yanariko (school yard), Achuta Grande (school yard) and Chambi Grande (school yard), and the presence of only one species of FLA in 4 samples (4/8, 50 %) (Table 3, Table 4 and Fig. 2).
d) In the cities of La Paz and El Alto, representing both urban areas, 6 samples (3 soil and 3 water) have been analyzed. The same 3 FLA species have been identified in those areas. *Acanthamoeba* spp. was present in 3 (50 %), of which 2 (33.3 %) were soil and 1 (16.7 %) were water samples; *B. mandrillaris* was present only in 1 (16.7 %) soil sample*;* and *V. vermiformis* was identified in all samples, both water and soil (6/6, 100 %). The coexistence of three and two FLA species was confirmed in La Paz, in the square of Mercado Lanza, and El Alto (Veterinary College - drainage canal), respectively and the presence of only one FLA species was confirmed in 3 samples (3/6, 50 %) (Table 3, Table 4 and Fig. 2).
e) One locality placed in a natural park in Cotapata and thus outside of the endemic area of fascioliasis, was also examined for FLA detection. *V. vermiformis* was identified in both, soil and water samples, and *Acanthamoeba* spp. was only identified in the soil sample. *B. mandrillaris* was not detected in any of the analyzed samples.

## 4 Discussion

The NBA is a hyperendemic region for human fascioliasis, where the highest reported prevalences and intensities of *Fasciola hepatica* infection in humans have been observed [[10], [11], [12], [13]]. New strategies have been introduced to reduce the risk of infection and re-infection, including the assessment of human infection sources to implement effective prevention measures, alongside a One Health initiative. However, it is important to consider that four animal species serve as reservoir for *F. hepatica* in this region; namely sheep, cattle, pigs, and donkeys, while lymnaeid snails play a crucial role in the transmission and epidemiology of fascioliasis [[31], [34]].

The presence of single genetically monomorphic introduced lymnaeid species, *Galba truncatula*, throughout the Bolivian Altiplano [29,38,43], simplifies research and control effort due to the lower complexity of transmission. Nevertheless, its ability to disperse passively complicates the geographical spread of this disease, as it could potentially spread on a large-scale due to livestock movement along the El Alto-Batallas/Peñas route [31]. Likewise, small-scale spread of lymnaeid may occur through the transport of goods and merchandise by donkeys, as well as through travel and movements between different localities or zones [32].

The NBA comprises endemic flat corridors between mountain ranges, at elevations of 3800–4100 m a.s.l. The high evapotranspiration at such extreme altitude prevents water from remaining for long periods after rainfall, which explains why the transmission foci in the Altiplano are mainly permanent [30], with human and animal infections occurring throughout the year. The flat corridors and extreme-altitude climate of the Northern Altiplano sharply contrast with other endemic regions where marked seasonal climate variations occur [44].

In addition to the numerous sources of human infection by *Fasciola hepatica* through the ingestion of metacercariae together with drinking water or aquatic plants [35], the presence of FLA in water and soil, and their correlation with the epidemiological characteristics of human fascioliasis in this endemic area of Bolivia, had never been studied. However, both diseases share the common factor that infections can occur in areas with a high prevalence of waterborne parasitic diseases within the human population [45,46].

In this work, 55 water and soil samples are analyzed for the first time for the detection of free-living amoebae in the 3 main corridors of the Northern Altiplano covering the main localities were human and animal fascioliasis has been confirmed from the 90s to the present. These localities are situated near human dwellings, communities and/or rural schools for children, health centers, among others [13], and recently proved to have expanded geographically [29]. The expansion of this endemic area is due to the presence of new foci with lymnaeid vectors outside the previously described limits, and which represent an expansion in altitudinal, northward and southward directions. This spread suggests an impact of increasing temperatures, by transforming previously unsuitable into suitable transmission habitats [15,29].

The distribution of FLA within this endemic area, and their abundant presence in all the localities studied in the 3 corridors, clearly suggests that FLA follow a dispersion/expansion pattern similar to that of the vectors of the fascioliasis, and that most likely have also been passively transported with the movements of humans and animals, or with food and merchandise.

When analyzing the localities in which the presence of the greatest number (up to three different species simultaneously) of FLA has been detected (as for instance in Chijipata Alta, Callucachi, Chiripaca, etc.) a great coincidence is found with the localities in which a greater prevalence of human or animal fascioliasis was reported (Chijipata Alta, 68.2–80 %, Callucachi, 72.0 % Chiripaca 84.6 %) [[10], [11], [12], [13]] as well as with specific localities that are highly frequented by humans such as health centres (Khanapata, Tiwanaku) or markets in the city of La Paz. This corroborates the relationship between the wide dispersion of these amoebae with human and animal activities and movements, similarly as in fascioliasis. It is evident that the high number of FLA in these localities represents a high risk for humans.

Among all the pathogenic FLA examined in this study, it is noteworthy that *Acanthamoeba* spp., *B. mandrillaris* and *V. vermiformis* have been detected in areas with high altitude, whereas *N. fowleri* does not appear to be able to survive under such extreme conditions. The genus *Acanthamoeba* and the species *V. vermiformis* are the most predominant in the three main corridors: Pucarani-Batallas that oscillate between 4001 and 3830 m, Tambillo-Huacullani (3871–3822 m) and Tiwanaku-Guaqui (3863–3847 m). The remarkable presence of FLA in this environment indicates its high adaptive capacity. In fact, *Acanthamoeba* could be isolated in mountain meadows at the very high altitudes of >4,100 m from Mila Mountain in Tibet [47]. In addition, *Acanthamoeba* is an opportunistic parasite, but, crucially, it is also regarded as an environmental biological quality indicator [48].

The species *V. vermiformis* is the most prevalent FLA, appearing in all samples collected in Bolivia (100 %). It reaches higher altitudes higher than those reported in other reports, such as in the snow on Teide volcano, where it was found at elevations between 2700 and 3718 m. This ubiquitous and thermotolerant amoeba is capable of spreading on its own and has been associated with pathogenic bacteria on several occasions [3,5,49]. Beside this, a recent study has reported that this amoeba is the etiological agent responsible for a clinical case of keratitis [50].

FLA infections caused by *Naegleria fowleri*, *Acanthamoeba* spp. or *V. vermiformis* are often related to water-related activities. In contrast, *B. mandrillaris* infections are typically linked to soil and dust exposure [3,51]. The infection route of *B. mandrillaris* remained unknown until 2003, when a case of granulomatous amoebic encephalitis (GAE) in a young girl in California, USA, led to the discovery of this pathogenic amoeba in a potted plant [52].

Finding *B. mandrillaris* in a cold, arid climate of the NBA is not unexpected, given that this organism has been identified worldwide, although it is more commonly reported in hotter climates. *B. mandrillaris* is an opportunistic FLA responsible for skin lesions and a type of encephalitis known as *Balamuthia* amoebic encephalitis (BAE). Granulomatous encephalitis caused by *B. mandrillaris* is a rare but highly lethal infection, with over 300 cases reported globally [[53], [54], [55]]. Most cases have been documented in the USA [56], Peru [57] and China [55].

Although *B. mandrillaris* has not typically been recorded in temperate regions, except for a reported human fatal case in Antofagasta, Chile [58], a location characterized by a coastal desert climate with no precipitation, high relative humidity, and minimal seasonal variation. Additional cases have been documented in temperate and subtropical zones, including Argentina [59,60] and Peru [61]. Notably, a single fatal case reported in the UK involved a patient who had recently traveled from Bolivia following r a motorcycle accident, which likely caused a small skin abrasion on the elbow [62].

In comparison to *Acanthamoeba*, *N. fowleri* and *V. vermiformis,* the detection of *B. mandrillaris* in environmental samples is significantly less frequent [52,57,[63], [64], [65], [66], [67], [68], [69], [70]]. Nonetheless, infections caused by *B. mandrillaris* are often associated with soil exposure [52,71,72]. Moreover, *B. mandrillaris* differs from other FLA in its laboratory growth requirements. This amoeba requires mammalian cell culture for successful growth, in contrast to other FLA, which can be cultured on agar plates coated with bacteria [56].

Taking into account that in all samples analyzed we were able to detect >1 to 10^2^ *Acanthamoeba, V. vermiformis and B. mandrillaris* cells (Ct value ≤35), this indicates that these FLAs are capable of surviving at extreme altitudes zones whose climates are cold and dry. This persistence may pose a potential health hazard to citizens residing in these rural and urban localities [73].

## 5 Conclusion

Our study demonstrates the presence of FLA in very high altitude areas such as the Northern Bolivian Altiplano and suggest that despite their ubiquitous nature, they have been able not only to adapt to these extreme environmental conditions but to expand in a way similar fascioliasis vectors, evidencing a parallelism in the dispersion of both parasitosis and serving to warn the health system of the risk of human infections by FLA in this hyperendemic area of fascioliasis, especially in human-associated environments.

## Funding

This research was funded by CIBER de Enfermedades Infecciosas (CIBERINFEC) Projects CB21/13/00056 and CB21/13/00100, ISCIII, Ministry of Science, Innovation and Universities, Madrid, Spain and European Union—NextGenerationEU; Project 2022CLISA26 “PROTCAN” from Fundación Cajacanarias/Fundación Bancaria la Caixa; and Projects No. 2016/099 and 2021/004 of the PROMETEO Program, Programa de Ayudas para Grupos de Investigación de Excelencia, Generalitat Valenciana, Valencia, Spain. PPP (TESIS2021010070) was funded by a grant from the Agencia Canaria de Investigación, Innovación y Sociedad de la Información de la Consejería de Economía, Conocimiento y Empleo, by Fondo Social Europeo (FSE) Programa Operativo Integrado de Canarias 2014–2020, Eje 3 Tema Prioritario 74 (85 %); OMGP and RLRE were also funded by Cabildo de Tenerife 2023–2028 (CC20230222, CABILDO.23). This research was also funded by VII Convocatoria de Proyectos de Cooperación al Desarrollo 2024, University of Valencia, Valencia, Spain (Project 2024/12).

## CRediT authorship contribution statement

**Patricia Pérez-Pérez:** Writing – review & editing, Methodology, Investigation, Formal analysis, Visualization. **Patricio Artigas:** Writing – original draft, Methodology, Investigation, Formal analysis, Data curation, Visualization, Conceptualization. **María Reyes-Batlle:** Visualization, Validation, Methodology, Data curation. **Elizabeth Córdoba-Lanús:** Validation, Supervision, Methodology, Investigation, Formal analysis. **Rubén L. Rodríguez-Expósito:** Methodology, Investigation, Formal analysis, Visualization. **Pablo F. Cuervo:** Resources, Methodology, Investigation, Visualization. **Angélica Domínguez-de-Barros:** Validation, Visualization, Formal analysis, Data curation. **Omar García-Pérez:** Validation, Supervision, Investigation, Formal analysis. **M. Adela Valero:** Resources, Methodology, Investigation, Project administration, Funding acquisition, Visualization. **Alejandra De Elías:** Resources, Methodology, Investigation, Visualization. **René Anglés:** Resources, Methodology, Visualization, Supervision. **Santiago Mas-Coma:** Writing – review & editing, Visualization, Validation, Supervision, Conceptualization. **José E. Piñero:** Writing – review & editing, Validation, Supervision, Resources, Methodology, Investigation, Visualization. **M. Dolores Bargues:** Writing – review & editing, Writing – original draft, Supervision, Resources, Methodology, Investigation, Formal analysis, Funding acquisition, Project administration, Data curation, Conceptualization. **Jacob Lorenzo-Morales:** Writing – review & editing, Writing – original draft, Validation, Supervision, Resources, Project administration, Methodology, Investigation, Funding acquisition, Formal analysis, Data curation, Conceptualization.

## Declaration of competing interest

The authors declare that they have no known competing financial interests or personal relationships that could have appeared to influence the work reported in this paper.

## Data Availability

Data will be made available on request.
